# Mirror movements in multiple sclerosis -a clinical, electrophysiological, and imaging study

**DOI:** 10.1186/s12883-024-03828-4

**Published:** 2024-09-06

**Authors:** Korbinian Holzapfel, Antonios Bayas, Markus Naumann, Tanupriya Ghosh, Verena Steuerwald, Martin Allweyer, Jan S. Kirschke, Lars Behrens

**Affiliations:** 1 Department of Neurology and Clinical Neurophysiology, Medical Faculty, University of Augsburg, Augsburg, Germany; 2 Department of Neuroradiology, Faculty of Medicine, Technical University of Munich, Munich, Germany; 3 Department of Diagnostic and Interventional Neuroradiology, Medical Faculty, University of Augsburg, Augsburg, Germany

**Keywords:** Mirror movements, Multiple sclerosis, Fatigue, Magnetic resonance imaging, Silent period

## Abstract

**Background:**

Mirror movements (MM) are commonly caused by a defect of interhemispheric pathways also affected in multiple sclerosis (MS), particularly the corpus callosum. We investigated the prevalence of MM in MS in relation to functional and morphological callosal fiber integrity by transcranial magnetic stimulation (TMS), magnetic resonance imaging (MRI), as well as fatigue.

**Methods:**

In 21 patients with relapsing–remitting MS and 19 healthy controls, MM were assessed and graded (Woods and Teuber scale: MM 1—4) using a bedside test. Fatigue was evaluated using the Fatigue Scale for Motor and Cognitive Functions (FSMC) questionnaire. TMS measured ipsilateral silent period latency and duration. MRI assessed callosal atrophy by measuring the normalized corpus callosum area (nCCA), corpus callosum index (CCI), and lesion volume.

**Results:**

MS patients had significantly more often and pronounced MM compared to healthy controls (*p* = 0.0002) and nCCA was significantly lower (*p* = 0.045) in MRI studies. Patients with higher MM scores (MM > 1 vs. MM 0/1) showed significantly more fatigue (higher FSMC sum score, *p* = 0.04, motor score, *p* = 0.01). In TMS and MRI studies, no significant differences were found between patients with MM 0/1 and those with MM > 1 (ipsilateral silent period measurements, CCA, CCI and lesion volume).

**Conclusions:**

MM are common in MS and can easily be detected through bedside testing. As MM are associated with fatigue, they might indicate fatigue in MS. It is possible that other cerebral structures, in addition to the corpus callosum, may contribute to the origin of MM in MS.

**Supplementary Information:**

The online version contains supplementary material available at 10.1186/s12883-024-03828-4.

## Background

Mirror movements (MM) refer to involuntary movements observed during voluntary activities in contralateral homologous muscles, particularly in the distal upper limb muscles [1, 2]. These movements are considered physiological during infancy but are observed in neurodegenerative diseases such as Parkinson’s disease [3], amyotrophic lateral sclerosis [4], and cerebrovascular disease [5, 6] as pathological finding. Additionally, enhanced mirror activity is observed in 'crossed' reaction time tasks in individuals with multiple sclerosis (MS) [7]. Different concepts regarding the origin of MM have been discussed [1]. Among them, the most prevailing is the interhemispheric dysfunctional connection, in which the commissural pathway via the corpus callosum plays a central role in the formation of MM. Published data suggest that damage to the corpus callosum in MS patients may lead to enhanced MM, which could be attributed to the loss of transcallosal inhibitory fibers [8].

Measuring the transcallosal inhibition (cortical silent period) via transcranial magnetic stimulation (TMS), is a method to investigate the functional integrity of callosal motor fibers [1, 9]. Changes in the ipsilateral silent period have been reported in people with MS [10]. Furthermore, structural abnormalities in the corpus callosum have been demonstrated via MRI in MS patients [11, 12]. Atrophy of the corpus callosum, particularly of the rostral part, has been observed and correlated with cognitive impairment and fatigue [13], which are common and debilitating symptoms of MS [14].

The aim of this study was to determine the prevalence of MM in patients with relapsing–remitting MS using a bedside test. Additionally, the study investigated the correlation between MM and fatigue (via the Fatigue Scale for Motor and Cognitive Functions, FSMC [15]), as well as morphological (MRI) and functional (TMS—cortical ipsilateral silent period) changes in callosal fiber integrity.

## Methods

The study included 21 patients with a diagnosis of relapsing–remitting MS according to McDonald criteria [16], treated at the Department of Neurology and Clinical Neurophysiology, University of Augsburg. To assess neurological impairment in participants with multiple sclerosis, the Expanded Disability Status Scale (EDSS) was used [17]. The exclusion criteria were as follows: age above 80 years, EDSS greater than 3.5, preexisting neurological diseases other than MS, and contraindication for TMS diagnostic testing (such as implanted metal in the body, history of seizures, or recent vertebral fracture).

Nineteen healthy individuals recruited from employees of the University Hospital Augsburg served as controls. All participants provided written informed consent to participate in the study.

The exclusion criteria of healthy controls were as follows: age above 80 years, preexisting neurological diseases, and contraindication for TMS diagnostic testing.

### MM

The presence of MM was evaluated by repetitively touching the thumb to alternate fingertips without any visual guidance in the voluntary hand while the contralateral arm remained rested on the lap. The Wood and Teuber scale was used to observe and assess the MM in the resting hand. The Wood and Teuber scale was developed in 1978 as an observation-based assessment [18] of MM and proved its reliability subsequently [19]. Prior to the study, we tested various movements and determined this task to be the most suitable for demonstrating MM. The subjects were asked to perform the task as neatly and quickly as possible with closed eyes for 10 consecutive repetitions without a break. The contralateral hand rested on the lap in a midpronated position with hand and fingers relaxed. The number of observed MM on the resting hand in relation to the contralateral being voluntarily moved was scored using the Wood and Teuber scale: 0—no clear imitative movement; 1—barely discernible movements; 2—slight MM or stronger but briefer repetitive movements; 3—strong and sustained repetitive movements; 4—movements equal to those expected from the intended hand.

The MM were assessed sequentially by two independent investigators for both hands in separate rooms, and the score of the hand with the highest rating on the Wood Teuber scale was used for further analysis in each subject.

### TMS (Ipsilateral silent period)

According to Hübers et al. [9], the ipsilateral silent period was studied in the abductor pollicis brevis (APB) muscle of each hand using a figure-of-eight shaped coil connected to a Magstim 200 stimulator (Magstim Company, Whitland, UK). The surface electromyogram (EMG) was recorded using disposable Ambu Neuroline 700 electrodes (Malaysia) with the belly-tendon technique. The EMG signal was band-pass filtered and digitized at a sampling rate of 50,000 Hz using a Nihon Kohden Neuropack S1 (Japan). The TMS stimulus was applied during maximal voluntary contraction of the APB, with the EMG signal serving as acoustic and visual feedback. All patients were able to perform a strong and maintaining contraction of the abductor pollicis brevis muscle. The coil was placed tangentially to the scalp, and the optimal stimulation site was determined as the location that produced the largest motor evoked potentials (MEPs) in the relaxed contralateral APB—known as the "sweet spot" [9]. The resting motor threshold was defined as the minimum intensity that elicited MEPs > 50 µV in at least 5 out of 10 consecutive trials according to Hübers et al. [9]. The TMS stimulus intensity was set at > 140% of the resting motor threshold. 10 Trials were performed. Latency and duration of the ipsilateral silent period were assessed for each individual trace where the ipsilateral silent period was clearly distinguishable, and averages were computed across all traces. The onset, latency, and duration of the ipsilateral silent period were measured for each hand.

### MRI

Cranial MRI images were obtained in MS patients with a magnetic field strength of 3 Tesla (Siemens Magnetom Vida, Siemens Healthineers, Erlangen, Germany) and in one case with 1.5 Tesla (Siemens Magnetom Avanto, Siemens Healthineers, Erlangen, Germany). All MRIs were performed using the same acquisition protocol, including a 3D fluid-attenuated inversion recovery (FLAIR) and a 3D spoiled gradient echo T1-weighted sequence (MPRAGE), both with an acquired isotropic voxel resolution of 1mm^3^. MRI images were evaluated by a senior neuroradiologist on the IntelliSpace platform (Philips Healthcare, Amsterdam, Netherlands).

Corpus callosum area (CCA) [11] and corpus callosum index (CCI) [12] were measured in the midsagittal plane of the 3D MPRAGE. CCA was measured as the midsagittal area by tracing the outer contour of the corpus callosum. Semiautomatic segmentation was used with manual tracing at the start of the outer contour and automatic completion of the segmentation. If necessary, the generated result was manually corrected if it did not follow the outer contour of the corpus callosum. CCA was normalized to head size. For this purpose, the midsagittal intracranial surface area was measured by semiautomatic segmentation of the inner skull lining with lower border in the plane of the foramen magnum. CCA was then divided by the intracranial surface area to obtain a normalized CCA (nCCA—%). CCI was calculated as the sum of the diameters of the genu, the splenium, and the middle part of the corpus callosum body divided by the greatest anteroposterior diameter of the corpus callosum from the anterior margin of the genu to the posterior margin of the splenium on the same midsagittal image. All these diameters were obtained by manual measuring (Supplementary Fig. 1). For the number of MS specific lesions all lesions in a juxtacortical/cortical, periventricular, or infratentorial location were counted. 10 MRI´s of healthy individuals from the Department of Neuroradiology, Augsburg, served as controls.

Lesion volume was determined in accordance with Pongratz et al. [20] by segmenting T2-hyperintense white matter lesions in the brain using the software LST (lesion segmentation tool, lesion growth algorithm) (https://www.applied-statistics.de/lst) and subsequently manually corrected by an experienced neuroradiologist. Lesions were classified as periventricular, (juxta)cortical or infratentorial according to current diagnostic criteria [16].

### Fatigue

To assess fatigue in MS participants, the Fatigue Scale for Motor and Cognitive Functions (FSMC) was used [15], yielding the FSMC sum score, FSMC motor scale, and FSMC cognitive scale.

### Statistical methods

GraphPad Prism 4 software was utilized for statistical analysis. To assess the agreement of the MM between two raters, Cohens’ Kappa Test was conducted. Spearman's correlation coefficient was used to examine the correlation of MM with the age and disease duration of the participants. Spearman's correlation coefficient was also employed to assess the correlation between patients' MM and TMS results (latency and duration of the ipsilateral silent period), as well as MRI measurements (CCI, CCA, total lesion volume, volume of periventricular, juxtacortical and infratentorial lesions). To compare the sex between patients and controls, the Chi-Square Test was used. The Mann–Whitney U-Test was performed to compare MM between patients and controls, as well as to compare the latency and duration of the ipsilateral silent period between patients and controls. Additionally, it was used to compare the ages of the patients and controls. Statistical significance was defined as *P* ≤ 0.05. Data are presented as the mean ± standard deviation (SD). For further analysis, we divided the patients with MM into two subgroups: MM 0/1 and MM > 1. The Mann–Whitney U-Test was employed to compare the FSMC between patient groups with MM 0/1 and MM > 1 (FSMC sum score, FSMC motor scale, FSMC cognitive scale), as well as to compare the EDSS, age, disease duration, and ipsilateral silent period measurements (latency and duration) of the two groups with MM 0/1 and MM > 1. In MRI studies, the Mann–Whitney U-Test was utilized to determine statistical differences in the CCI and CCA between patients and controls, as well as between patient groups with MM 0/1 vs MM > 1, and for lesion volumes between patient groups with MM 0/1 vs MM > 1 as well.

## Results

The study enrolled 21 relapsing–remitting MS patients with an EDSS ranging from 0 to 3.5 (mean 1.5 ± 1.2) and 19 healthy volunteers.

Table 1 presents clinical and demographic data of patients and healthy controls.

**Table 1:** presents clinical and demographic data of patients and healthy controls

Parameters	Patients (*n* = 21)	Controls (*n* = 19)
Sex (male); n (%)	6 (28.57)	5 (26.31)
Age (years)	31.8 ± 12.7	35.3 ± 11.1
Disease duration (years)	4.4 ± 6.4	NA
EDSS	1.5 ± 1.2	NA
FSMC sum score	49.3 ± 14.7	NA
FSMC motor scale	23.2 ± 8.1	NA
FSMC cognitive scale	26.2 ± 7.1	NA

Demographic and clinical data of the participants

Age, disease duration, EDSS, FSMC sum score, FSMC motor scale and FSMC cognitive scale are presented as mean ± standard deviation, unless otherwise indicated. Sex is presented as number (percentage). n, number of subjects; NA, not applicable

There were no significant differences between patients and controls regarding age and sex (*p* = 0.58; *p* = 0,56).

**Fig. 1:**
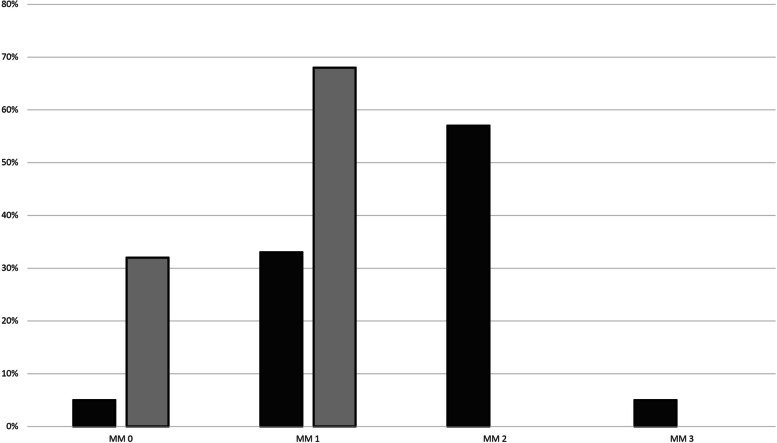
Mirror Movements (Wood and Teuber scale). MM, mirror movements; %, percentage of patients (black)/controls (grey)

Figure 1 shows MM according to the scale of Woods and Teuber from 0—3, in patients and healthy controls (patients: MM 0 *n* = 1, MM 1 *n* = 7, MM 2 *n* = 12, MM 3 *n* = 1; controls: MM 0 *n* = 6, MM 1 *n* = 13, MM > 1 *n* = 0).

There was a high level of agreement between the two raters (kappa = 0.80) for MM testing using the Woods and Teuber scale.

Significant differences were found between controls and patients regarding the extent of MM manifestation (Woods and Teuber patients/controls: mean 1.6 ± 0.7 vs 0.7 ± 0.5; *p* = 0.0002).

MM did not correlate with age or disease duration in MS patients (*p* = 0.97, *p* = 0.82). However, there was a moderate positive correlation between EDSS and FSMC sum score (*r* = 0.53, *p* = 0.02), FSMC cognitive scale (*r* = 0.54, *p* = 0.01), and FSMC motor scale (*r* = 0.46, *p* = 0.04).

In TMS studies, there was no significant difference between patients and controls in the latency (patients, mean 34.7 ± 3.7 ms; controls, mean 33.4 ± 5.1 ms; *p* = 0.51) and duration (patients, mean 24.1 ± 4.3 ms; controls, mean 24.0 ± 4.1 ms; *p* = 1.00) of the ipsilateral silent period. MM also exhibited no correlation with the latency and duration of the ipsilateral silent period in MS patients (*p* = 0.92, *p* = 0.14). Three participants (two patients, one control) had no optimal stimulation site on the scalp (sweet spot) detected, so no ipsilateral silent period could be investigated.

Patients with MM > 1 had a significantly higher FSMC sum score (mean 55.50 ± 14.59 vs 40.00 ± 9.17; *p* = 0.04), FSMC motor score (mean 29.67 ± 6.08 vs 21.00 ± 5.26; *p* = 0.01), and a trend for a higher FSMC cognitive score (mean 25.83 ± 8.99 vs 19.13 ± 4.39; *p* = 0.15) than patients with MM 0/1.

There were no significant differences in age (*p* = 0.80), disease duration (*p* = 0.44), or EDSS (*p* = 0.28) between the two groups (MM 0/1 vs MM > 1).

**Fig. 2:**
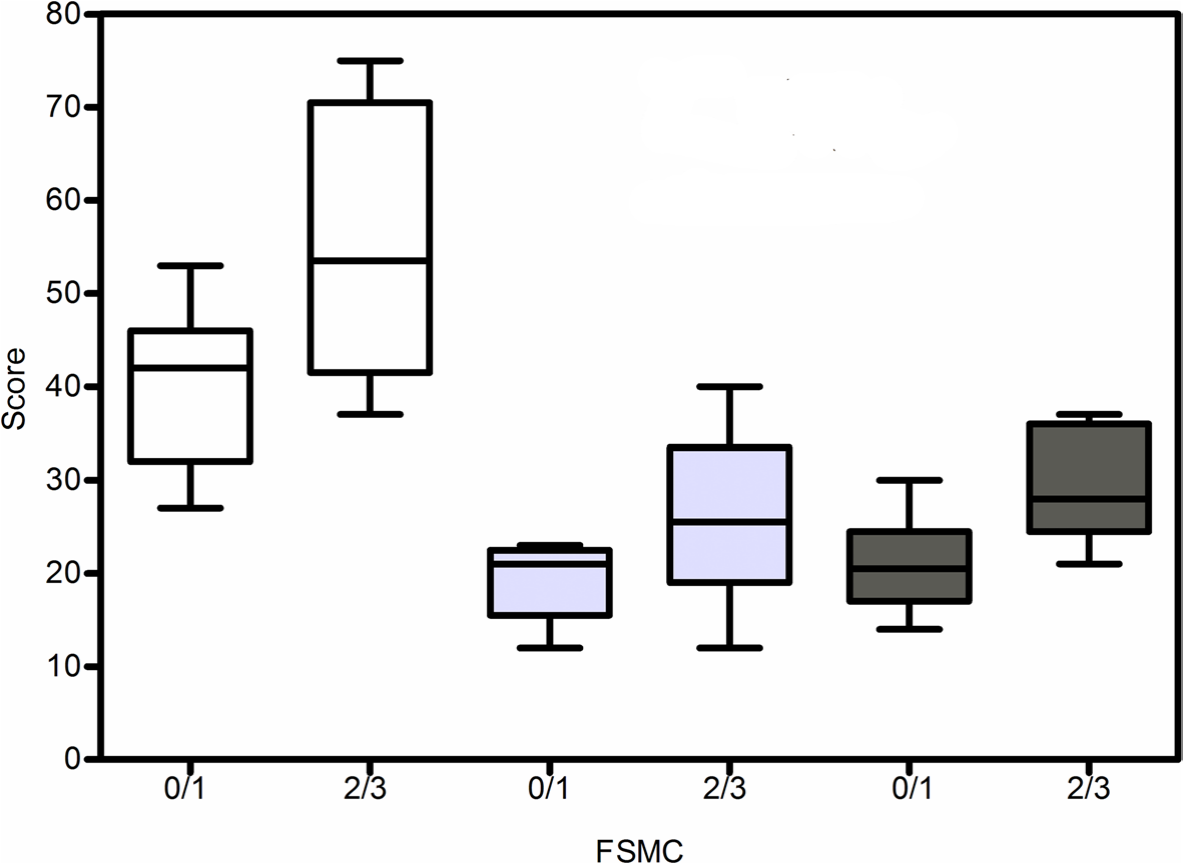
Boxplots of the FSMC sum score, motor score, and cognitive score. 0/1, patients with MM 0/1; 2/3, patients with MM > 1; FSMC, Fatigue Scale for Motor and Cognitive Functions; Median (line at the middle), 25th to 75th percentile (box) and range (error bars) are shown. Left—sum score; middle—cognitive score; right—motor score

Figure 2 shows boxplots of the FSMC sum score, motor score, and cognitive score.

In TMS studies, no significant differences were observed in the latency (*p* = 0.97) and duration (*p* = 0.17) of the ipsilateral silent period between patients with MM 0/1 and those with MM > 1.

NCCA was significantly lower in patients than in controls (mean 3.53 ± 0.74% vs 4.32 ± 0.62%; *p* = 0.015), and CCI was numerically lower (mean 36.37 ± 8.01% vs 42.03 ± 3.60%; *p* = 0.055). MM showed no correlation with CCI or CCA in MS patients (*p* = 0.67, *p* = 0.75), nor did it exhibit any correlation with the total lesion volume, the periventricular (*p* = 0.28), juxtacortical (*p* = 0.23) or infratentorial volume (*p* = 0.05).

There were no significant differences in CCA (MM 0/1 vs. MM > 1 mean 5.41 ± 0.67 cm^2^ vs 5.55 ± 1.39 cm^2^; *p* = 0.56) or CCI (MM 0/1 vs. MM > 1 mean 36.46 ± 4.87% vs 36.32 ± 9.65%; *p* = 0.97) between patient groups with MM 0/1 and MM > 1.

In patients with MM > 1, the total lesion volume (MM 0/1 vs MM > 1 mean 1.33 ± 1.54 cm^3^ vs 6.59 ± 9.45 cm^3^; *p* = 0.33) as well as the volume of periventricular (MM 0/1 vs MM > 1 mean 0.85 ± 1.09 cm^3^ vs 5.29 ± 8.53 cm^3^; *p* = 0.26) and juxtacortical lesions (MM 0/1 vs MM > 1 mean 0.26 ± 0.28 cm^3^ vs 0.79 ± 0.95 cm^3^; *p* = 0.37) was larger compared to patients with MM 0/1, but did not reach statistical significance.

There were no infratentorial lesions in the MM 0/1group, the mean number in the MM > 1 group was 0.14 ± 0.33 cm^3^.

## Discussion

In our study, we assessed MM in mildly to moderately affected patients with relapsing–remitting MS (EDSS ≤ 3.5 (mean 1.5 ± 1.2)) and compared them with healthy controls.

Our results indicated that MS patients had more frequently and pronounced MM compared to healthy controls. The high interrater agreement, in line with Magne et al. [19], emphasized the usability of the Woods and Teuber scale for assessing MM.

We selected mildly to moderately affected MS patients since previous examinations had shown that severely affected patients with paralytic, spastic, or atactic debilitations either would not be able to perform the investigation for MM, or the MM would be masked due to the patients’ existing debilitations. Furthermore, TMS studies were not possible in more severely affected patients due to our TMS protocol, which requires maximum voluntary contraction of the APB.

Our study focused on MM as a clinical sign and reliably detected and quantified MM in MS patients and controls through bedside testing. Testing MM through bedside testing provided sufficient information for our study; however, a limitation of our study is that we did not perform additional EMG recordings of MM for further quantification and objectification of MM.

The FSMC is a patient-reported outcome measure for measuring mental and physical fatigue in MS patients [15]. In our study, there was a moderate correlation between all three FSMC scores and the EDSS. Patients with higher scores of MM on the Woods Teuber Scale (> 1) had significantly higher FSMC sum scores and motor scales, as well as numerically higher scores in the FSMC cognitive scale, compared to patients with lower scores. Studies with larger numbers of patients should be conducted to determine whether our results are confirmed and whether a significant difference on the FSMC cognitive scale is achieved between the two groups (MM 0/1 vs. > 1). There was no significant difference in age, duration of the disease, or EDSS between the two groups, ruling out these parameters as confounding factors.

A possible explanation for the correlation of MM and motor fatigue in MS patients may be that both symptoms may origin in similar brain areas, involving neuronal dysfunction and reorganization. Several studies [14, 21, 22] have shown altered cerebral activation in MS-related fatigue, with increased activation required for performing motor tasks in MS patients with fatigue, including the cingulate gyri [23], that also showed overactivation in Parkinson patients with MM [3]. Overactivation of the (posterior) cingulate cortex might be part of a network disruption in MS patients, leading to an inability to inhibit MM.

We did not find a correlation between MM and the age of patients or the duration of the disease, since age and disease duration (mean age 31.8 ± 12.7 years, mean disease duration 4.4 ± 6.4 years) were quite homogenously distributed. In patients with longer disease duration or elderly patients, bedside testing of MM may be constrained due to disability (e.g. spasticity) or age-related limitations interfering with MM testing. Whether results may be different in older patients and those with a longer disease duration may be subject to further studies.

About 70% of healthy controls in our study exhibited mild MM (MM = 1). MM in healthy individuals are also described in the literature, and only the involuntary synkinetic MM of the opposite limb should be considered pathologic [1]. Our study did not identify any MM > 1 in the control group, consistent with the literature. Mayston et al. [2] however, were unable to detect subtle MM (MM = 1) in adults using EMG, despite employing the same test as in our study. In our study, participants performed the testing with their eyes closed, as we had observed increased occurrences of subtle MM prior to the study in the absence of visual control. Mayston et al. [2] did not document whether the testing was conducted with eyes open or closed in their study, so their results cannot be compared easily to ours.

In our TMS studies, we investigated the ipsilateral silent period according to Hübers et al. [9]. Our results were in line with the normal values documented in their study. There was no significant difference in the latency and duration of the ipsilateral silent period between patients and controls in our study and no correlation was found between MM and the latency and the duration of the ipsilateral silent period in MS patients. Tataroglu et al. [10] found a prolongation of the ipsilateral silent period duration in 78% of their patients with definite MS who were more severely affected (mean EDSS 2.8 vs. 1.5 in our study) and investigated the ipsilateral silent period in the legs but did not analyze it separately in the arms. Some patients with higher EDSS scores and the presence of paralysis/spasticity may not be able to undergo ipsilateral silent period investigation through maximal voluntary contraction according to our protocol. Therefore, we decided to study upper extremity movements, often less affected especially in patients with spinal lesions.

One aim of our study was to investigate the morphology of the corpus callosum in MS patients with MM, as it is the most important commissural structure. We examined the CCA and CCI in patients and controls to determine possible corpus callosum atrophy. Consistent with a study by Granberg et al. [11], we found significant reductions in CCA in MS patients, and a non-significant reduction in CCI. It should be noted that patients in the Granberg study were more severely affected and had a longer disease duration than those in our study.

We did not observe any differences in CCI or CCA between the groups with MM 0/1 and MM > 1 using MRI and we found no correlation between MM and CCI and CCA.

A possible explanation for the increased MM in MS patients may be altered functional pathways in the corpus callosum due to strategically located demyelinating plaques, resulting in interhemispheric inhibition dysfunction even before any morphological changes in commissural fibers are established. Cabib et al. [7] investigated mirror activity in MS patients using EMG and found enhanced EMG activity in contralateral homologous muscles, as well as significant diffusivity changes in the corpus callosum via diffusion tensor imaging in MRI studies, not measured in our routine MRI. Similarly, in ALS patients with MM, Wittstock et al. [24] did not find diffusion changes in the corpus callosum despite prolongation of latency or loss of the ipsilateral silent period in MEP studies, suggesting that disruptions in transcallosal pathways, as measured through TMS, may precede microstructural alterations in the corpus callosum. In contrast, in our study we observed a co-occurrence of increased MM and morphological changes in the corpus callosum (reduced nCCA) in MS patients, which precedes functional changes measured by TMS, not different between patients and controls.

Similarly to our negative TMS findings, Jung et al. [25] found in their study on MS patients, where they investigated the ipsilateral silent period as a marker for callosal demyelination, no correlation between changes in the ipsilateral silent period (particularly the duration of the ipsilateral silent period) and lesion volume in the corpus callosum or callosal atrophy (low sensitivity of the ipsilateral silent period at 28% despite a high proportion of patients (78%) with visible CC lesions in MRI). Their explanation, which could also apply to our results, was that the duration of the ipsilateral silent period likely reflects demyelination, while MRI measurements are more indicative of inflammation (T2w lesions) or axonal degeneration (MRI measures of atrophy).

Other structures than the corpus callosum may contribute to the origin of MM in MS patients. Liu et al. [1] reported a deactivation of a non-mirroring inhibitory network in Parkinson patients (dorsolateral prefrontal cortex, presupplementary motor area) – areas that may also be affected by juxtacortical inflammatory lesions in MS patients. Tisseyre et al. [26] were able to demonstrate mirror contractions in the EMG of patients with pyramidal tract involvement in spinal injuries. These patients also exhibited decreased interhemispheric coherence, which may reflect reduced interhemispheric inhibition. In our study, some patients exhibited MS-lesions in the spinal cord which might also lead to reduced interhemispheric inhibition as well.

However, spinal MRIs were not evaluated in our study. Further studies are warranted to address this aspect. Functional magnetic resonance imaging (fMRI) studies in poststroke patients [27] have reported increased activity in the non-lesioned sensorimotor cortex, with MM in the non-paretic hand attributed to activity in the contralesioned sensorimotor area via the crossed corticospinal tract—a possible mechanism in patients with MM. We observed a non-significantly higher volume of juxtacortical and periventricular lesions in patients with MM > 1 compared to patients with MM 0/1. Statistical significance might be achieved through a study with a larger number of patients.

It would be of interest whether lesion volume in specific areas (e.g. the dorsolateral prefrontal cortex and the presupplementary motor area) may contribute to MM. However, no specific cortical areas causing MM have been identified so far.

## Conclusion

In our study, we were able to demonstrate that using the Woods and Teuber scale for bedside testing is a viable method for detecting and quantifying MM in MS patients. This approach is easily implemented and can provide valuable insights.

We could show that MM are prevalent among mildly to moderately affected MS patients, with an EDSS score up to 3.5. Additionally, we identified an association between MM and fatigue, particularly motor fatigue.

Since fatigue as a frequent complaint in MS is often quantified by self-assessment lacking objective testing, further longitudinal studies are necessary to explore whether MM might serve as an objective marker for fatigue.

Our study has some limitations. We focused on a limited sample of MS patients who were mildly to moderately affected. As mentioned above, it may provide further insights to include a larger number of patients with more severe disabilities and longer disease duration, yet still retaining preserved hand function, in future investigations.

Additional research, including fMRI of the brain and positron emission tomography (PET), exploring cortical and subcortical structures beyond the corpus callosum, may acquire a more comprehensive understanding of MM resulting from neuronal disruption in MS.

## Supplementary Information

Supplementary Material 1.

## Data Availability

Data is available upon reasonable request.

## Abbreviations

APB: Abductor pollicis brevis
CCA: Corpus callosum area
CCI: Corpus callosum index
EDSS: Expanded Disability Status Scale
EMG: Electromyogram
FLAIR: Fluid-attenuated inversion recovery
fMRI: Functional magnetic resonance imaging
FSMC: Fatigue Scale for Motor and Cognitive Functions
LST: Lesion segmentation tool
MEPs: Motor evoked potentials
MM: Mirror movements
MPRAGE: Magnetization Prepared Rapid Acquisition with Gradient Echoes
MRI: Magnetic resonance imaging
MS: Multiple sclerosis
nCCA: Normalized corpus callosum area
PET: Positron emission tomography
SD: Standard deviation
TMS: Transcranial magnetic stimulation
