# Supplementary material for: Mirror movements in multiple sclerosis -a clinical, electrophysiological, and imaging study
Source: BMC Neurol. 2024 Sep 6;24:326. doi: 10.1186/s12883-024-03828-4 (PMC11378473; doi:10.1186/s12883-024-03828-4)
Supplement: Supplementary file 1 — Supplementary Material 1. [file 12883_2024_3828_MOESM1_ESM.docx]

**Supplementary Figure 1**


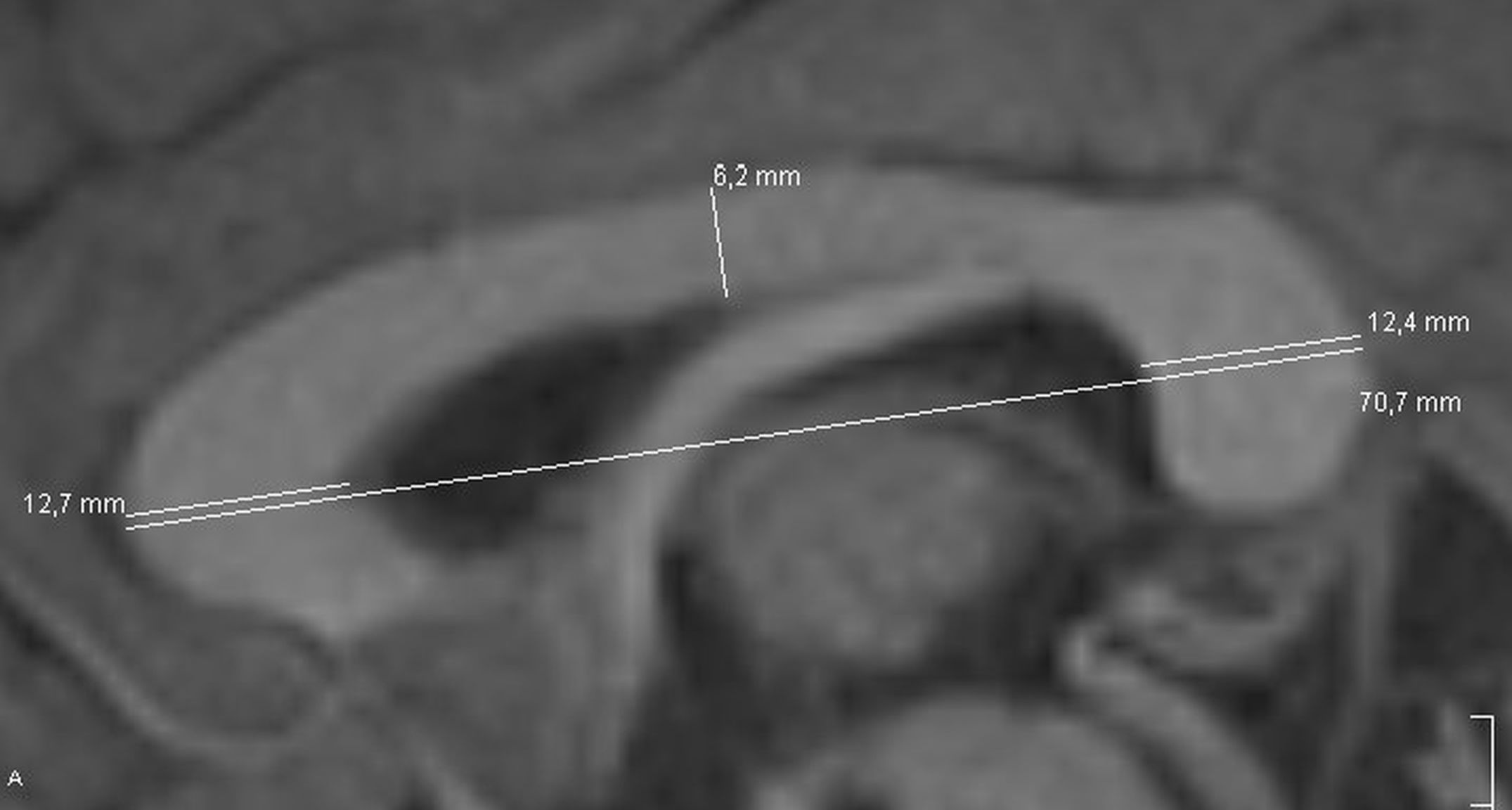


Example of the measurements to calculate CCI with diameters of the genu (12.7 mm), the splenium (12.4 mm), middle part of the corpus callosum (6.2 mm) and greatest anteroposterior diameter (70.7 mm).
